# Contribution to the Study of Hydrastis Canadensis

**Published:** 1888-04

**Authors:** 


### *CONTRIBUTION TO THE STUDY OF HYDRASTIS CANADENSIS.*

Givopiszew, of St. Petersburg, has recently made an elaborate study of this old American remedy, with the following results:

1. Aqueous extracts of hydrastis, even in large doses, are not poisonous to warm-blooded animals.

2. Hydrastis always produces cardiac depression and consequent reduction of arterial tension.

3. It always produces uterine contractions. The aqueous extract is to be preferred for this purpose. The contractions of the pregnant uterus near term are most powerful, those of the virgin uterus weakest.

4. Large doses of hydrastis may induce premature labor after the fourth month.

The author sums up the clinical uses of hydrastis as follows:

1. Hydrastis is an excellent remedy for uterine hemorrhages due to inflammations or misplacements of this organ; also for profuse hemorrhages occurring about the menopause.

2. The uterine contractions produced by hydrastis are weaker than those produced by ergot.

3. The use of this drug is followed by no untoward symptoms. It produces no gastro-intestinal disturbance, but, on the contrary, will frequently relieve dyspepsia.—*Bulletin Gen. de Therapeutique.*

